# Development of Attenuated Total Reflectance Mid-Infrared (ATR-MIR) and Near-Infrared (NIR) Spectroscopy for the Determination of Resistant Starch Content in Wheat Grains

**DOI:** 10.1155/2021/5599388

**Published:** 2021-07-14

**Authors:** Rong Wang, Xia Wei, Hongpan Wang, Linshu Zhao, Cengli Zeng, Bingrui Wang, Wenying Zhang, Luxiang Liu, Yanhao Xu

**Affiliations:** ^1^Hubei Key Laboratory of Waterlogging Disaster and Agriculture Use of Wetland and Hubei Collaborative Innovation Centre for Grain Industry and Engineering Research Center of Ecology and Agriculture Use of Wetland, Ministry of Education, Yangtze University, Jingzhou, Hubei 434025, China; ^2^Hubei Key Laboratory of Food Crop Germplasm and Genetic Improvement, Food Crops Institute, Hubei Academy of Agricultural Sciences, Wuhan 430064, China; ^3^Institute of Crop Sciences, Chinese Academy of Agricultural Sciences, Beijing 100081, China; ^4^Hubei Engineering Research Center for Protection and Utilization of Special Biological Resources in the Hanjiang River Basin, Jianghan University, Wuhan 430056, China; ^5^College of Plant Science & Technology, Huazhong Agricultural University, Wuhan 430064, China

## Abstract

The chemical method for the determination of the resistant starch (RS) content in grains is time-consuming and labor intensive. Near-infrared (NIR) and attenuated total reflectance mid-infrared (ATR-MIR) spectroscopy are rapid and nondestructive analytical techniques for determining grain quality. This study was the first report to establish and compare these two spectroscopic techniques for determining the RS content in wheat grains. Calibration models with four preprocessing techniques based on the partial least squares (PLS) algorithm were built. In the NIR technique, the mean normalization + Savitzky–Golay smoothing (MN + SGS) preprocessing technique had a higher coefficient of determination (*R*_*c*_^2^ = 0.672; *R*_*p*_^2^ = 0.552) and a relative lower root mean square error value (RMSEC = 0.385; RMSEP = 0.459). In the ATR-MIR technique, the baseline preprocessing method exhibited a better performance regarding to the values of coefficient of determination (*R*_*c*_^2^ = 0.927; *R*_*p*_^2^ = 0.828) and mean square error value (RMSEC = 0.153; RMSEP = 0.284). The validation of the developed best NIR and ATR-MIR calibration models showed that the ATR-MIR best calibration model has a better RS prediction ability than the NIR best calibration model. Two high grain RS content wheat mutants were screened out by the ATR-MIR best calibration model from the wheat mutant library. There was no significant difference between the predicted values and chemical measured values in the two high RS content mutants. It proved that the ATR-MIR model can be a perfect substitute in RS measuring. All the results indicated that the ATR-MIR spectroscopy with improved screening efficiency can be used as a fast, rapid, and nondestructive method in high grain RS content wheat breeding.

## 1. Introduction

Resistant starch (RS) is the starch that cannot be converted into glucose when passing through the healthy small intestine [1]. Owing to its indigestion, RS can increase satiety and reduce calorie intake, which could reduce postprandial blood glucose levels [2], regulate the intestinal metabolism [3], reduce colon cancer risk [4], control bodyweight [5], and absorb minerals [6]. Due to its benefits to human health, the RS studies have attracted considerable attentions and promoted to be one of the important discoveries about the relationship between carbohydrates and human health [1, 2, 7]. It is also becoming a hot topic for function food breeding [7–9].

Starch in grains is the major source of carbohydrates in the human diet. The improvement of the RS content in grains is an important goal for breeding. A few high RS content grain varieties, such as RS111 [10], the hulless barley variety Himalaya 292 [11], and durum wheat [12], have been released to the public. While, they still cannot satisfy the growing demand. Induced mutagenesis and selection of natural mutations are still the major approaches for the breeding of high RS varieties [7, 13–17]. Currently, enzyme hydrolysis and the chromogenic method are the commonly used methods for RS measurement. These methods are destructive, time-consuming, costly, and cumbersome, thus delaying the process of research in RS [18]. A simple, fast, and nondestructive method for screening mutants with millions of mutants becomes vital for breeding.

Infrared spectroscopy, including near-infrared (NIR, 950–1650 nm) and attenuated total reflectance mid-infrared (ATR-MIR, 525–4000 cm^−1^) spectroscopy, has been widely used as a simple, fast, and reliable substitute to conventional methods in discrimination of chemical composition [19–21]. MIR spectra can identify the fundamental vibrational absorption of functional groups in the mid-infrared region (525–4000 cm^−1^), while the NIR spectra in the range of 950 nm and 1650 nm identify overtone information and combinations of these vibrations [19–21]. The NIR technique was adopted as an official method for the prediction of crude proteins in wheat grains by the American Association of Cereal Chemists (AACC) [22]. A model based on NIR spectroscopy has been expanded to the investigation of the amylose content [23], lipid content [24], water content [25], and deoxynivalenol content in durum wheat [26] and for monitoring the wheat gluten enzyme [27]. Meanwhile, the MIR spectroscopy models have been used to analyze sugars in barley [19, 28], ash and moisture content in soybean [24], and proteins and lipids in different wheat varieties [29], as well as to perform nitrate determination in paddy soil [30].

Wheat (*Triticum aestivum* L.) feeds about 40% of the world's population [31]. Wheat grains with the high RS content could provide additional health benefits [8, 9, 31]. While, there is no spectroscopy model for determining the content of RS in wheat grains yet. Furthermore, mutation breeding is the major approach for the breeding of high RS varieties [13–17]. However, there is still no simple, fast, and nondestructive method for screening high RS mutants in breeding.

The aims of this study are to develop a simple, fast, nondestructive method for the determination of the RS content in wheat grains by using NIR and ATR-MIR spectroscopy and to apply the developed spectral methods to screen the high grain RS content wheat mutants.

## 2. Materials and Methods

### 2.1. Samples

Based on the rule of the range of the RS content in the calibration set should cover those in the validation set, a total of sixty-four (*n* = 64) wheat accessions were randomly divided into a calibration set (fifty-one wheat samples, Table 1) and a validation set (thirteen wheat samples, Table 2) in a ratio of 4 : 1. The calibration set and the validation set were used to develop and validate the best calibration model for the prediction of the RS content in wheat grains by NIR and ATR-MIR approaches.

The *M*_5_ generation wheat mutant library, including 1010 mutant lines, was used to screen the high RS content mutant lines with the best calibration model from the above results. The mutants originated from wheat accession YUW-1-207 and were irradiated by a 50 Gy ^7^Li ion beam.

All wheat materials were grown at Yangtze University field stations in 2017-2018. The field trial experiments were arranged randomized with three replications for each accession. Each replicate was designed 1.2 m long and 0.85 m wide. The seeding density was kept 30 per row. Fertilization, pest, and disease control were performed on a regular basis. The analysis was only based on the plants in the middle row.

### 2.2. Chemical Measured RS Content

Whole grain flour was prepared from each sample by grinding in a pulveriser (Perten Laboratory Mill 3100), which was fitted with a 0.8 mm screen. The RS content (the amount of RS as a percentage of whole grain) was measured for 100 mg whole grain flour using a resistant starch assay kit (K-RSTAR, Megazyme Co., Wicklow, Ireland) following the manufacturer's instructions. The sample was treated with 10 mg/mL pancreatic a-amylase and 3 U/mL amyloglucosidase (AMG) enzymes for hydrolysis and solubilization of nonresistant starch. After the enzymatic reaction was terminated by adding a 50% ethanol solution, resistant starch was recovered as a pellet by centrifugation (approx. 4000 r/min, 10 min). Resistant starch in the pellet was dissolved in 2 M KOH before the reacted solution was repeatedly washed and decanted. Then, starch in the solution was quantitatively hydrolyzed to glucose with AMG. D-glucose was measured with glucose oxidase/peroxidase (GOPOD) reagent at 510 nm wavelength against the reagent blank [29]. All samples were measured with three replicates. The standard RS sample from the reagent kit was used as a control in each round of reactions.

### 2.3. NIR and ATR-MIR Spectroscopy

NIR spectra were collected in the range between 950 nm and 1650 nm using a DA7200 spectrometer (Perten Instruments Inc., Sweden). In the NIR method, approximately 4 g of wheat grains per sample was scanned in triplicate in a small ring cup. Each spectrum represented the average of 32 scans and was recorded as log (1/R) at 2 nm increments.

MIR spectra were scanned based on a Nicolet iS5 Fourier transform infrared spectrometer (ThermoFisher Scientific, USA) with the iD7 attenuated total reflectance (ATR) accessory. The ATR-MIR spectra of each sample were obtained by taking the means of 16 scans at a resolution of 4 cm^−1^, in the range between 525 and 4000 cm^−1^, with a background of 16 scans. The air was recorded as a reference background spectrum. The ATR crystal was carefully cleaned with ethanol after each sample measurement.

### 2.4. Data Analysis

The NIR and ATR-MIR spectra were uploaded to Unscrambler 9.7 software (CAMO Corporation, USA) for chemometric analysis. The models for the calibration between the measured values and the infrared spectra were established using partial least squares (PLS) regression with full cross-validation. The quality of the models was assessed by the determination coefficient of calibration (*R*_*c*_^2^), the determination coefficient of prediction (*R*_*p*_^2^), the root mean square error of calibration (RMSEC), and the root mean square error of prediction (RMSEP) [32]. Moreover, the residual predictive deviation (RPD), a statistical parameter defined as the ratio of the standard deviation (SD) to the RMSEP [30, 32], was used to assess the predictive ability of the calibration models.

The preprocessing methods were used to eliminate the interferences of background signal, random noise, and light scattering from the spectra, which can be divided into the scatter correction group and spectral derivatization group [33, 34]. Four preprocessing methods including Gaussian filter smoothing (GFS), multiplicative scatter correction (MSC), baseline mean normalization (MN), and Savitzky–Golay smoothing (SGS) were used to transform the NIR and ATR-MIR spectra before calibration to eliminate interference noise such as baseline drift, tilt and reverse, and light scattering [35, 36]. The MN, baseline, and MSC preprocessing methods belonged to the scatter correction group, and the SGS and GFS belong to the spectral derivatization group. The developed model, with the highest value of determination coefficient, the lowest value of root mean square error (RMSE), and the highest value of RPD, was chosen as the best calibration model.

## 3. Results

### 3.1. Chemical Measured Wheat Grain RS Content

The RS content in whole wheat grain flour samples was measured by the AOAC method in the calibration set ranged from 0.220% to 3.348% with the mean content 1.011% (Table 1); while the RS content in the validation set ranged from 0.267% to 2.842% with the mean content 1.285% (Table 2). The standard deviation (SD) in the calibration set and validation set is 0.679 and 0.697, respectively. The coefficient of variation (CV) in the calibration set and validation set is 67.086% and 53.846%, respectively. A wide distribution of the wheat grain RS content was observed in the calibration set and validation set.

### 3.2. Development of NIR and ART-MIR Prediction Models

**Figure 1:**
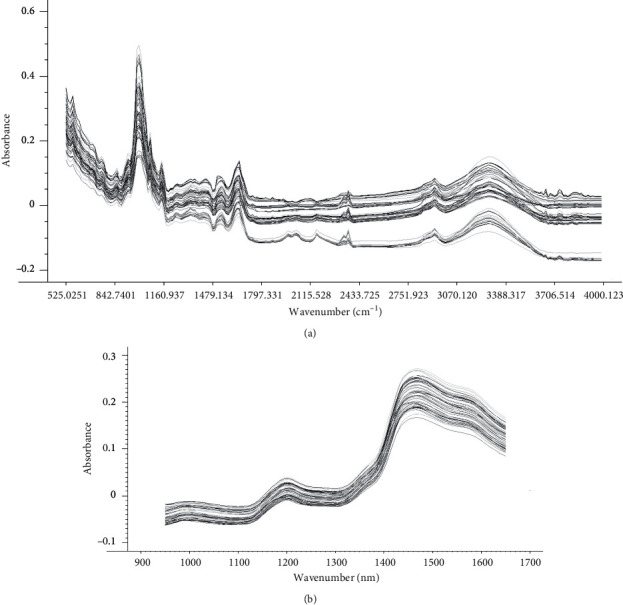
ATR-MIR and NIR spectra of wheat grain samples obtained in this study. (a) ATR-MIR spectra obtained in the range between 525 and 4000 cm^−1^ without pretreatment. (b) NIR spectra obtained in the range between 950 and 1650 nm without pretreatment.

The ATR-MIR spectrum (525–4000 cm^−1^) (Figure 1(a)) region has strong absorption peaks and belongs to the fundamental molecular vibration modes. The peaks between 3600 and 3000 cm^−1^ were assigned to hydrogen bonded water (O-H stretching vibration). The weak band detected at 1652 cm^−1^ was responsible for C=O vibration of the decarboxylated groups. The region between 1200 and 950 cm^−1^ was attributed to the O-C stretch vibrations of the glucose ring [37, 38]. 1146 cm^−1^ and 1047 cm^−1^ are the stretching vibrations linked to the primary and secondary alcohol hydroxyl groups in glucose, and 1002 cm^−1^ is the C-O stretching vibration of the pyranose ring. C-O-H, C-C-H, and O-C-H bending of the anomeric configuration of carbohydrates occurred between 750 and 950 cm^−1^ [39, 40]. For the NIR spectra (950–1650 nm) (Figure 1(b)), two peaks were observed; the weak intensity was found round 1200 nm, and the intense peak was found around 1500 nm. The absorption around 1215 nm and 1483 nm was, respectively, reported to be related to the stretching of C-H and N-H [41].

The collected ATR-MIR and NIR spectra from the calibration set were used to develop calibration models through PLS regression with full cross-validation. For the NIR spectra, the model with MN + SGS preprocessing had the highest *R*_*c*_^2^ and RPD, which were 0.672 and 1.464, respectively. At the same time, RMSE in this model reached as low as 0.459 (Table 3). The MN + SGS preprocessing was chosen as the best calibration model for the NIR spectroscopy. For the ATR-MIR spectra, the baseline preprocessing method was chosen as the best calibration model with *R*_*c*_^2^, RPD, and RMSE 0.937, 2.391, and 0.284, respectively (Table 3).

**Figure 2:**
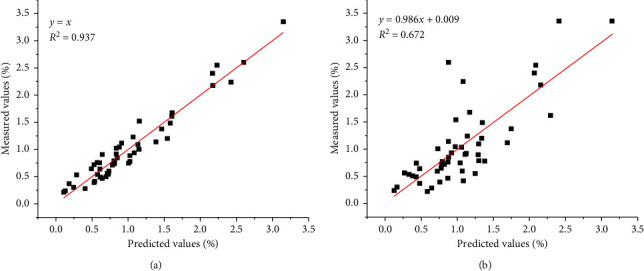
Relation between the measured values and the predicted values for the grain resistance starch content by the calibration models obtained by ATR-MIR and NIR. (a) The calibration model obtained by ATR-MIR. (b) The calibration model obtained by NIR.

Meanwhile, a much better correlation of the chemical determined values and the predicted values was observed in the ATR-MIR spectroscopy model (Figure 2(a)) than in the NIR spectroscopy model (Figure 2(b)). The correlation between predicted values and real values in the ATR-MIR model was 0.937, while the value in the NIR model was 0.672. The results showed the ATR-MIR spectroscopy model may have a better performance in the prediction of the RS content in wheat grains than the NIR spectroscopy model.

### 3.3. Validation of NIR and ATR-MIR Best Calibration Models

**Figure 3:**
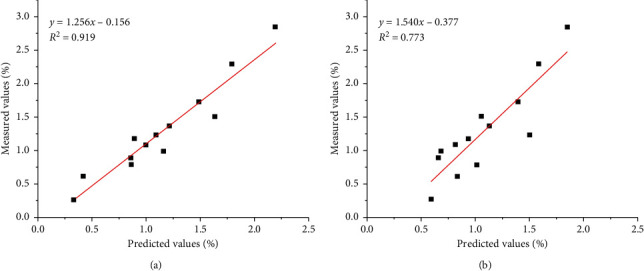
The external validation of the best ATR-MIR and NIR models. (a) The ATR-MIR model using the baseline preprocessing method. (b) The NIR model using the MN + SGS pretreatment method.

To verify the accuracy and repeatability of models with the best preprocessing techniques in two spectroscopies, validation was performed in the validation set. The experimentally determined RS values, the predicted RS values, and the calculated relative error (measurement/prediction value) are given in Table 2. For the NIR calibration model with the MN + SGS preprocessing, the relative error ranged from 17.143% to 121.348%, with a mean relative error of 34.028%. For the ATR-MIR calibration model with the baseline preprocessing method, the relative error ranged from 2.931% to 31.220%, with a mean relative error of 15.832%. A linear regression analysis was performed between the measured value and the value predicted by the ATR-MIR model (*R*^2^ = 0.919) (Figure 3(a)) and the NIR model (*R*^2^ = 0.773) (Figure 3(b)). The results confirmed that the ATR-MIR spectroscopy provided a better performance than NIR spectroscopy for the prediction of the RS content in wheat grains.

### 3.4. Screen of High RS Wheat Mutants by the ATR-MIR Model

**Figure 4:**
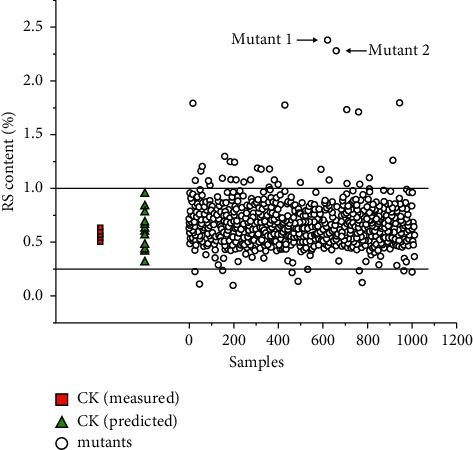
Application of the best ATR-MIR model to screen the high grain RS content mutant lines from wheat mutant library.

To check the application of the developed ATR-MIT model, the best ATR-MIR calibration model with baseline preprocessing was promoted to predict the grain RS content of 1010 wheat mutants (Figure 4). The predicted RS content of 1010 wheat mutants ranged from 0.101 ± 0.018% to 2.553 ± 0.311%. Two lines with YUW-RSH1 (2.553 ± 0.311%) and YUW-RSH2 (2.116 ± 0.230%) highest RS content were identified (Table 4). At the same time, the RS content was also validated by the chemical method. The chemical determined RS content in YUW-RSH1 and YUW-RSH2 was 2.572 ± 0.090% and 2.126 ± 0.071%, respectively (Table 4). There was no significant difference between the predicted values and chemical determined values. The results showed that ATR-MIR spectroscopy can be an effective way to screen and identify high grain RS content materials for wheat breeding.

## 4. Discussion

### 4.1. ATR-MIR Spectroscopy

*Had a Better Performance for the Prediction of the RS Content in Wheat Grains than NIR Spectroscopy. *The MIR and NIR spectroscopies have become the fastest growing and most compelling modern quantitative analysis technologies for assessment of the quality of both macronutrients and minor compounds in agricultural products and food [21, 27, 42, 43]. In agriculture, protein, tannins, lipids, phytic acid, and most of the amino acids were the commonly detected components in crops [23, 27–29, 34]. In this study, a total of 64 wheat samples were used to develop the ATR-MIR and NIR calibration models for the prediction of the RS content in wheat grains. Compared with previous studies, the samples and the distribution of the wheat grain RS content in this study have reached the requirements of infrared basic modeling [20, 24, 42].

It is commonly accepted that regression models with *R*^2^ above 0.91 are regarded as excellent, *R*^2^ = 0.82–0.9 indicated good predictive ability; *R*^2^ = 0.50–0.65 reveals approximate quantitative performance [44]; and when RPD was greater than 2, the models were considered excellent, whereas values lower than 1.5 indicate not enough for applications [45]. In our results, the best NIR models showed an approximate quantitative performance (*R*_*c*_^2^ = 0.672; *R*_*p*_^2^ = 0.552; RPD = 1.464), and the best MIR model gave a good prediction performance (*R*_*c*_^2^ = 0.927; *R*_*p*_^2^ = 0.828; RPD = 2.366). Overall, the ATR-MIR displayed a better performance for the evaluation of the wheat grain RS content.

Previous reports comparing the ATR-MIR and NIR techniques for the measurement of chemical differences and quantitative analysis of substances showed that the NIR and MIR techniques have different prediction effects [21, 24, 42, 46]. In soybean samples, the NIR technique was suggested for the prediction of protein and lipid determination, while the MIR technique was suggested for ash and moisture determination [24]. In rice samples, the NIR technique and the MIR technique were the best predictors of starch and protein, respectively [46]. In this study, we found that the ATR-MIR spectroscopy had a better performance for the prediction of the RS content in wheat grains than NIR spectroscopy based on the PLS regression. Nowadays, the recent developed deep learning and artificial intelligence algorithm could be used to improve the stability and robustness of the spectral model, which may also become trend of the future research [47].

### 4.2. Rapid RS Content Assessment Method for Wheat Breeding

To date, mutation breeding is the major approach for the breeding of high RS varieties [7, 13–17]. Rapid quality assessment methods are taking an increasing important role in breeding programs; this is especially the case in mutation breeding. In this study, the developed best calibration ATR-MIR model was used to screen out 2 high resistance starch wheat mutants from the mutant library. Comparing with the chemical methods with 24 hours' time cost for each sample, the total time for the spectroscopic method just needs less than five minutes per sample, which provided the possibility to screening tens of thousands of breeding materials. The spectroscopic method could greatly improve the measurement efficiency and provide a new approach for crop resistant starch breeding and research.

## 5. Conclusion

MIR and NIR techniques were demonstrated to be useful for the prediction of the RS content in wheat grains. The ATR-MIR technique provided a better predictive ability than the NIR technique. The total time required for the measurement of each sample was less than 5 minutes, compared to approximately 20 hours required for the determination of the RS content by chemical methods. In addition, we confirmed that the use of ATR-MIR spectroscopy to assist in the screening and identification of the wheat RS content was an effective approach.

## Figures and Tables

**Table 1:** Wheat samples in the calibration set and the grain RS content measured by the chemical method.

Wheat samples	Grain RS content (%)
Shannong7859	0.220 ± 0.013
Karagan	0.242 ± 0.011
Baiqimai	0.288 ± 0.013
Fan6	0.308 ± 0.018
Mace	0.373 ± 0.020
Fretes-3	0.395 ± 0.021
Mazhamai	0.417 ± 0.025
Dabaimai	0.474 ± 0.022
Ganmai8hao	0.491 ± 0.025
Huaimai16	0.504 ± 0.032
Zhongyou16	0.536 ± 0.031
Youzimai	0.541 ± 0.032
Lumai1hao	0.549 ± 0.033
Honglidangnianlao	0.562 ± 0.034
Sumai3hao	0.597 ± 0.041
Xinmai19	0.606 ± 0.043
Jinmai2148	0.642 ± 0.042
Mingxian169	0.648 ± 0.045
Yanzhan4110	0.718 ± 0.056
Jinan2hao	0.721 ± 0.053
Jinan17hao	0.741 ± 0.052
Jinan13	0.750 ± 0.054
Geerhongmai	1.677 ± 0.084
Zijiehong	2.177 ± 0.103
Shuilizhan	2.238 ± 0.152
Muzongzhuoga	2.399 ± 0.112
Shite14	0.758 ± 0.052
Kord CL Plus	0.758 ± 0.055
Xuzhou21	0.761 ± 0.061
Sankecun	0.780 ± 0.063
Zheng6fu	0.793 ± 0.062
Diyouzao	0.847 ± 0.065
N553	0.892 ± 0.061
Kopara 73	0.910 ± 0.062
Honghuamai	0.919 ± 0.076
Yunmai34	0.938 ± 0.075
Miannong4hao	1.008 ± 0.075
Meiqianwu	1.046 ± 0.073
Jinmai8hao (Jinzhong849)	1.082 ± 0.076
Ningmai9hao	1.091 ± 0.071
Wenmai6hao (Yumai49)	1.119 ± 0.078
Jiahongmai	1.139 ± 0.071
AUS 19399	1.200 ± 0.072
Liuyuehong	1.232 ± 0.083
Jiangmai	1.379 ± 0.083
Huzhuhong	1.487 ± 0.083
Zang2726	1.533 ± 0.086
Honghuazao	1.615 ± 0.083
Heshangmai	2.548 ± 0.107
Wujiangzhuo	2.601 ± 0.155
Baihuamai	3.348 ± 0.167

**Table 2:** Wheat samples in the validation set and grain RS content determined by both the chemical method and the best ATR-MIR and NIR calibration models.

Wheat samples	Grain RS content (%)			Relative error (RE)^*∗*^	
	Chemical measured	ATR-MIR predicted	NIR predicted	ATR-MIR RE	NIR RE
Xiaokouhong	0.267 ± 0.011	0.332 ± 0.041	0.591 ± 0.085	24.345	121.348
Yu30691-1-3	0.615 ± 0.032	0.423 ± 0.032	0.834 ± 0.088	31.212	35.610
Hongpidongmai	0.784 ± 0.041	0.864 ± 0.065	1.019 ± 0.091	10.204	29.974
Tanori	0.887 ± 0.063	0.861 ± 0.073	0.661 ± 0.093	2.931	25.479
Hongpixiaomai	0.991 ± 0.068	1.160 ± 0.082	0.684 ± 0.082	17.053	30.979
PI94365	1.028 ± 0.065	0.998 ± 0.081	0.814 ± 0.091	2.918	20.817
Huadong6hao	1.172 ± 0.072	0.892 ± 0.093	0.937 ± 0.105	23.891	20.051
Tumangmai	1.230 ± 0.083	1.093 ± 0.101	1.506 ± 0.132	11.138	22.439
Mahon Demias	1.365 ± 0.085	1.216 ± 0.097	1.131 ± 0.139	10.916	17.143
Chixiaomai	1.508 ± 0.095	1.637 ± 0.105	1.054 ± 0.108	8.554	30.106
Baitiaoyu	1.725 ± 0.105	1.487 ± 0.127	1.398 ± 0.153	13.797	18.957
Mangxiaomai	2.288 ± 0.112	1.798 ± 0.153	1.587 ± 0.186	21.416	30.638
Lanhuamai	2.842 ± 0.117	2.200 ± 0.194	1.851 ± 0.225	22.590	34.870

^*∗*^RE, the ratio between the measured value minus the predicted value divided by the measured value.

**Table 3:** Develop and screening for the best calibration model for resistant starch in wheat grain samples using ATR-MIR and NIR spectra.

Spectroscopy	Preprocessing methods	Calibration		Internal cross-validation		
		*R*_*c*_^2^	RMSEC	*R*_*p*_^2^	RMSEP	RPD
NIR	Original	0.641	0.403	0.482	0.493	1.363
	MN	0.673	0.384	0.526	0.472	1.424
	MN + MSC	0.671	0.385	0.523	0.474	1.418
	MN + SGS	0.672	0.385	0.552	0.459	1.464

ATR-MIR	Original	0.922	0.188	0.804	0.303	2.218
	Baseline	0.937	0.169	0.828	0.284	2.366
	Baseline + GFS	0.935	0.171	0.826	0.286	2.35
	Baseline + SGS	0.935	0.171	0.825	0.287	2.341

*R*_*c*_^2^, determination coefficient of calibration; *R*_*p*_^2^, determination coefficient of prediction; RMSEC, root mean square error of calibration; RMSEP, root mean square error of prediction; RPD, residual predictive deviation; MN, mean normalization; MSC, multiplicative scatter correction; SGS, Savitzky–Golay smoothing; GFS, Gaussian filter smoothing.

**Table 4:** Verification of the best ATR-MIR calibration model screened high grain RS wheat mutants by the chemical measured method.

	YUW-RSH1		YUW-RSH2	
	ATR-MIR predicted (%)	Chemical measured (%)	ATR-MIR predicted (%)	Chemical measured (%)
Replication 1	2.623	2.487	2.378	2.121
Replication 2	2.245	2.66	1.945	2.199
Replication 3	2.791	2.569	2.025	2.058
Variance analysis	2.553 ± 0.311a	2.572 ± 0.090a	2.116 ± 0.230a	2.126 ± 0.071a

## Data Availability

Majority of the data used to support the findings of this study are included within the article. Other data are made available from the first author and corresponding authors upon request.

## References

[1] Genoni A., Christophersen C. T., Lo J. (2020). Long-term Paleolithic diet is associated with lower resistant starch intake, different gut microbiota composition and increased serum TMAO concentrations. *European Journal of Nutrition*.

[2] Kumar A., Sahoo U., Baisakha B. (2018). Resistant starch could be decisive in determining the glycemic index of rice cultivars. *Journal of Cereal Science*.

[3] Hald S., Schioldan A. G., Moore M. E. (2016). Effects of arabinoxylan and resistant starch on intestinal microbiota and short-chain fatty acids in subjects with metabolic syndrome: a randomised crossover study. *PLoS One*.

[4] Yuan H., Zhu X., Chen D., Wang W., Meng S., Wang J. (2017). Effects of dual modified resistant indica rice starch on azoxymethane-induced incipient colon cancer in mice. *Experimental and Therapeutic Medicine*.

[5] Keenan M. J., Zhou J., McCutcheon K. L. (2006). Effects of resistant starch, A non-digestible fermentable fiber, on reducing body fat. *Obesity*.

[6] Raigond P., Ezekiel R., Raigond B. (2015). Resistant starch in food: a review. *Journal of the Science of Food and Agriculture*.

[7] Petropoulou K., Salt L. J., Edwards C. H. (2020). A natural mutation in Pisum sativum L. (pea) alters starch assembly and improves glucose homeostasis in humans. *Nature Food*.

[8] Lafiandra D., Riccardi G., Shewry P. R. (2014). Improving cereal grain carbohydrates for diet and health. *Journal of Cereal Science*.

[9] Hansson S. O., Åman P., Becker W. (2018). Breeding for public health: a strategy. *Trends in Food Science & Technology*.

[10] Yang C. Z., Shu X. L., Zhang L. L. (2006). Starch properties of mutant rice high in resistant starch. *Journal of Agricultural and Food Chemistry*.

[11] Topping D. L., Morell M. K., King R. A., Li Z., Bird A. R., Noakes M. (2003). Resistant starch and health-himalaya 292, a novel barley cultivar to deliver benefits to consumers. *Starch—Stärke*.

[12] Hazard B., Zhang X., Naemeh M., Dubcovsky J. (2014). Registration of durum wheat germplasm lines with combined mutations in SBEII a and SBEIIb genes conferring increased amylose and resistant starch. *Journal of Plant Registrations*.

[13] Botticella E., Sestili F., Hernandez-Lopez A., Phillips A., Lafiandra D. (2011). High resolution melting analysis for the detection of EMS induced mutations in wheat SbeIIa genes. *BMC Plant Biology*.

[14] Hazard B., Zhang X., Colasuonno P. (2012). Induced mutations in the starch branching enzyme II (SBEII) genes increase amylose and resistant starch content in durum wheat. *Crop Science*.

[15] Shu X., Xu J., Wang Y., Rasmussen S. K., Wu D. (2013). Effects of gamma irradiation on starch digestibility of rice with different resistant starch content. *International Journal of Food Science & Technology*.

[16] Mishra A., Singh A., Sharma M., Kumar P., Roy J. (2016). Development of EMS-induced mutation population for amylose and resistant starch variation in bread wheat (*Triticum aestivum*) and identification of candidate genes responsible for amylose variation. *BMC Plant Biology*.

[17] Schönhofen A., Zhang X., Dubcovsky J. (2017). Combined mutations in five wheat STARCH branching enzyme II genes improve resistant starch but affect grain yield and bread-making quality. *Journal of Cereal Science*.

[18] Perera A., Meda V., Tyler R. T. (2010). Resistant starch: a review of analytical protocols for determining resistant starch and of factors affecting the resistant starch content of foods. *Food Research International*.

[19] Cozzolino D., Roumeliotis S., Eglinton J. (2014). Feasibility study on the use of attenuated total reflectance MIR spectroscopy to measure the fructan content in barley. *Analytical Methods*.

[20] Cozzolino D., Degner S., Eglinton J. (2014). A novel approach to monitor the hydrolysis of barley (hordeum vulgare L) malt: a chemometrics approach. *Journal of Agricultural and Food Chemistry*.

[21] Carbas B., Machado N., Oppolzer D. (2020). Comparison of near-infrared (NIR) and mid-infrared (MIR) spectroscopy for the determination of nutritional and antinutritional parameters in common beans. *Food Chemistry*.

[22] Osborne B. G., Fearn T., Hindle P. H. (1993). Practical NIR spectroscopy with applications in food and beverage analysis. *Book Practical NIR Spectroscopy with Applications in Food and Beverage Analysis*.

[23] Sampaio P. S., Soares A., Castanho A., Almeida A. S., Oliveira J., Brites C. (2018). Optimization of rice amylose determination by NIR-spectroscopy using PLS chemometrics algorithms. *Food Chemistry*.

[24] Ferreira D. S., Galão O. F., Pallone J. A. L., Poppi R. J. (2014). Comparison and application of near-infrared (NIR) and mid-infrared (MIR) spectroscopy for determination of quality parameters in soybean samples. *Food Control*.

[25] Torres I., Sánchez M.-T., Benlloch-González M., Pérez-Marín D. (2019). Irrigation decision support based on leaf relative water content determination in olive grove using near infrared spectroscopy. *Biosystems Engineering*.

[26] Lippolis V., Pascale M., Cervellieri S., Damascelli A., Visconti A. (2014). Screening of deoxynivalenol contamination in durum wheat by MOS-based electronic nose and identification of the relevant pattern of volatile compounds. *Food Control*.

[27] Zhang Y., Luo L., Li J. (2017). In-situ and real-time monitoring of enzymatic process of wheat gluten by miniature fiber NIR spectrometer. *Food Research International*.

[28] Huang Y., Carragher J., Cozzolino D. (2016). Measurement of fructose, glucose, maltose and sucrose in barley malt using attenuated total reflectance mid-infrared spectroscopy. *Food Analytical Methods*.

[29] Sujka K., Koczoń P., Ceglińska A., Reder M., Ciemniewska-Żytkiewicz H. (2017). The application of FT-IR spectroscopy for quality control of flours obtained from polish producers. *Journal of Analytical Methods in Chemistry*.

[30] McCleary B. V., McNally M., Rossiter P. (2002). Measurement of resistant starch by enzymatic digestion in starch and selected plant materials: collaborative study. *Journal of AOAC International*.

[31] Hazard B., Trafford K., Lovegrove A., Griffiths S., Uauy C., Shewry P. (2020). Strategies to improve wheat for human health. *Nature Food*.

[32] Shao Y., Du C., Zhou J. (2017). Quantitative analysis of different nitrogen isotope labelled nitrates in paddy soil using mid-infrared attenuated total reflectance spectroscopy. *Analytical Methods*.

[33] Barnes R. J., Dhanoa M. S., Lister S. J. (1989). Standard normal variate transformation and de-trending of near-infrared diffuse reflectance spectra. *Applied Spectroscopy*.

[34] Geladi P., MacDougall D., Martens H. (1985). Linearization and scatter-correction for near-infrared reflectance spectra of meat. *Applied Spectroscopy*.

[35] McGoverin C. M., Clark A. S. S., Holroyd S. E., Gordon K. C. (2010). Raman spectroscopic quantification of milk powder constituents. *Analytica Chimica Acta*.

[36] Almeida M. R., Oliveira K. d. S., Stephani R., de Oliveira L. F. C. (2011). Fourier-transform Raman analysis of milk powder: a potential method for rapid quality screening. *Journal of Raman Spectroscopy*.

[37] Damiran D., Yu P. (2011). Molecular basis of structural makeup of hulless barley in relation to rumen degradation kinetics and intestinal availability in dairy cattle: a novel approach. *Journal of Dairy Science*.

[38] Zhang X., Yu P. (2012). Using ATR-FT/IR molecular spectroscopy to detect effects of blend DDGS inclusion level on the molecular structure spectral and metabolic characteristics of the proteins in hulless barley. *Spectrochimica Acta Part A: Molecular and Biomolecular Spectroscopy*.

[39] Stuart B., Ando D. J. (1996). Modern infrared spectroscopy: analytical chemistry by open learning. *Book Modern Infrared Spectroscopy: Analytical Chemistry by Open Learning*.

[40] Grassi S., Amigo J. M., Lyndgaard C. B., Foschino R., Casiraghi E. (2014). Assessment of the sugars and ethanol development in beer fermentation with FT-IR and multivariate curve resolution models. *Food Research International*.

[41] Xie L., Ye X., Liu D., Ying Y. (2009). Quantification of glucose, fructose and sucrose in bayberry juice by NIR and PLS. *Food Chemistry*.

[42] Shi H., Lei Y., Louzada Prates L., Yu P. (2019). Evaluation of near-infrared (NIR) and fourier transform mid-infrared (ATR-FT/MIR) spectroscopy techniques combined with chemometrics for the determination of crude protein and intestinal protein digestibility of wheat. *Food Chemistry*.

[43] Rossi G. B., Lozano V. A. (2020). Simultaneous determination of quality parameters in yerba mate (Ilex paraguariensis) samples by application of near-infrared (NIR) spectroscopy and partial least squares (PLS). *LWT-food Science and Technology*.

[44] Williams P., Antoniszyn J. (2019). *Near-Infrared Technology: Getting the Best Out of Light*.

[45] Smyth H. E., Cozzolino D., Cynkar W. U., Dambergs R. G., Sefton M., Gishen M. (2008). Near infrared spectroscopy as a rapid tool to measure volatile aroma compounds in Riesling wine: possibilities and limits. *Analytical and Bioanalytical Chemistry*.

[46] Shao Y., Cen Y., He Y., Liu F. (2011). Infrared spectroscopy and chemometrics for the starch and protein prediction in irradiated rice. *Food Chemistry*.

[47] Shin S., Lee Y., Kim S., Choi S., Kim J. G., Lee K. (2021). Rapid and non-destructive spectroscopic method for classifying beef freshness using a deep spectral network fused with myoglobin information. *Food Chemistry*.

